# miR-3934 regulates the apoptosis and secretion of inflammatory cytokines of basophils via targeting RAGE in asthma

**DOI:** 10.1186/s13223-022-00704-z

**Published:** 2022-08-04

**Authors:** Liyan Dou, Wenyu Wang, Junwei Wang, Xiaofei Zhang, Xiaoman Hu, Weili Zheng, Kaiyu Han, Guangyou Wang

**Affiliations:** 1 Department of Cardiology, The Fourth Affiliated Hospital of Harbin Medical University, Heilongjiang, China; 2 Department of Respiratory and Critical Medicine, The Second Affiliated Hospital of Harbin Medical University, Heilongjiang, China; 3 Department of Neurobiology, Harbin Medical University, Heilongjiang, China

**Keywords:** Asthma, miR-3934, Basophils, Inflammatory cytokines, TGF-β/Smad signaling pathway

## Abstract

**Background:**

Several miRNAs are now known to have clear connections to the pathogenesis of asthma. The present study focused on the potential role of miR-3934 during asthma development.

**Methods:**

miR-3934 was detected as a down-regulated miRNA in basophils by sequencing analysis. Next, the expression levels of miR-3934 in peripheral blood mononuclear cells of 50 asthma patients and 50 healthy volunteers were examined by RT-qPCR methods. The basophils were then treated with AGEs and transfected with miR-3934 mimics. The apoptosis levels were examined by flow cytometry assay; and the expression levels of cytokines were detected using the ELISA kits. Finally, the Western blot was performed to examined the expression of key molecules in the TGF-β/Smad signaling pathway.

**Results:**

miR-3934 was down-regulated in the basophils of asthmatic patients. The expression of the pro-inflammatory cytokines IL-6, IL-8 and IL-33 was enhanced in basophils from asthmatic patients, and this effect was partially reversed by transfection of miR-3934 mimics. Furthermore, receiver operating characteristics analysis showed that miR-3934 levels can be used to distinguish asthma patients from healthy individuals. miR-3934 partially inhibited advanced glycation end products-induced increases in basophil apoptosis by suppressing expression of RAGE.

**Conclusion:**

Our results indicate that miR-3934 acts to mitigate the pathogenesis of asthma by targeting RAGE and suppressing TGF-β/Smad signaling.

## Introduction

Asthma is a common respiratory disease characterized by chronic airway inflammation and hyperresponsiveness; its symptoms include recurrent wheeze, shortness of breath, chest tightness and cough [1]. Asthma affects more than 270 million people, including both adults and children, and its prevalence is rising worldwide [2]. About 3–10% of all cases of adult asthma are severe [3, 4]. These patients usually need a high dose of controller medication and frequent reliever therapy, and face the risk of acute attack, despite optimized treatment [5]. This makes severe asthma a serious health concern and economic burden globally [6–8]. Nevertheless, the mechanisms underlying the pathogenesis of asthma are not entirely clear.

microRNAs (miRNA) are a family of small noncoding RNAs that act by suppressing expression of their target genes [9–11]. Several miRNAs reportedly exhibit clear connections to the pathogenesis of asthma. These include microRNA-1248 (miR-1248) [12], miR-1291 [13], miR-570-3p [10], and let-7a [14], which are known to be expressed in asthma patients’ lungs. Because a single miRNA can simultaneously target different genes [15], these asthma-related miRNAs are involved in the occurrence of asthma in various ways, including regulating the cytodifferentiation of T cells, macrophages and alveolar epithelial cells, and promoting the synthesis and secretion of asthma relevant cytokines [16]. However, there are still multiple miRNAs whose role in the pathogenesis of asthma is still unclear.

Basophils are thought to participate in the early asthmatic response, as they are associated with the synthesis and secretion of histamine and cysteinyl leukotrienes, both of which are involved in bronchoconstriction [17–19]. Recent studies have also shown that basophils play an essential role in promoting delayed airway inflammation [20, 21]. We previously observed that advanced glycation end products (AGEs) can increase apoptosis among basophils and are also closely associated with increased secretion of inflammatory cytokines [22]. Receptor for advanced glycation end products (RAGE) is a type I AGE receptor and is involved in the occurrence of multiple chronic lung diseases, including asthma [22, 23]. Activated basophils express their receptor RAGE on their surface upon induction by AGEs. In the present study, therefore, we focused on the relationship among miRNAs, RAGE and basophils in asthma in an effort to identify novel therapeutic approaches to the diagnosis of asthma.

## Material and methods

### Patients and clinical tissue samples

The present study involved a total of 50 patients with asthma hospitalized at the Second Affiliated Hospital of Harbin Medical University between January 2018 and September 2019. The diagnosis of asthmatics met the American Thoracic Society refractory asthma definition [24]. The control group included 50 healthy volunteers. Controls had no history of lung disease, allergy, respiratory infection or exposure to tobacco smoke. The clinical characteristics of all subjects are summarized in Table 1. Each patient signed informed consent regarding the trial. The ethical committee of the Second Affiliated Hospital of Harbin Medical University approved this study.

**Table 1:** Clinical characteristics of the asthma patients and controls

	Asthma patients (n = 50)	Controls (n = 50)	P value
Gender—n (%) (Male/female)	33(66%)/17(34%)	29(58%)/21(42%)	0.169
Age-median (IQR)	30(21,44)	36(23–49)	0.42
Smoking history—n (%) (Yes/No)	35(70%)/15(30%)	0(0%)/50(100%)	< 0.001
Allergy history—n (%) (Yes/No)	8(16%)/42(84%)	0(0%)/50(100%)	< 0.001
White blood cell -median (IQR) (normal range 4.0–10.0 × 10^9/L)	6.3(3.7–8.0)	7.2(4.6–9.5)	0.548
Eosinophil-median (IQR) (normal range 0.05–0.45 × 10^9/L)	0.99(0.34–1.77)	0.36(0.12–0.50)	0.11

### Isolation of human basophils

Venous blood was obtained from consenting healthy volunteers and subjects with asthma. Basophils were semi-purified by means of density centrifugation using Percoll solution as previously described [25]. Briefly, Percoll solutions with two different densities (1.079 and 1.070 g/ml) were used. The blood samples were centrifugated at room temperature for 15 min at 700 × *g*, after which cells in the layer between the two Percoll solutions were further purified by negative selection using an EasySep Human Basophil Kit (StemCell Technologies, CAN) according to the manufacturer’s instructions. The yield was 1–3 × 10^4^ basophils/ml of peripheral \jblood, depending on the donor. Fluorescence activating cell sorter results showed the mean basophil purity to be consistently > 98%, and the viability was consistently > 94%. AGEs were prepared by adding 5 g of bovine serum albumin and 9 g of D-glucose to 100 mL PBS at 37 °C for 8 h. The basophils were the treated with 400 mg/L AGEs.

### Transfection

miR-3934 mimics, miR-3934 mimics negative control (NC) and a RAGE overexpression plasmid were synthesized by Shanghai GenePharma Co., Ltd (Shanghai, China). Using lipofectamine 3000 (Invitrogen) according to the manufacturer’s protocol, basophils were transfected with miR-3934 mimics or miR-3934 mimics NC with or without the RAGE overexpression plasmid. Reverse transcriptase quantitative real-time PCR (RT-qPCR) methods were used to determine transfection efficiency.

### RT-qPCR

Total RNAs were extracted from cells using TRIzol reagent (Invitrogen) and reversed transcribed into cDNAs using PrimeScript RT Master Mix (Takara, Dalian, China). qPCR was then performed to detect the expression levels of miR-3934 in different samples using SYBR premix Ex Taq (Takara) on an ABI 7500 qPCR System (Applied Biosystems Life Technologies, Foster City, USA). Relative expression levels of miRNA-3934 were determined using the 2^−ΔΔCt^ method and were normalized to U6. The PCR protocol entailed 95 °C for 30 s followed by 40 cycles of 95 °C for 5 s and 60 °C for 30 s. The primers were synthesized by Sangon Biotech (Shanghai) Co., Ltd (Shanghai, China). The primers used for real-time PCR were as follows: RAGE forward primer: 5′-GTGTCCTTCCCAACGGCTC-3′, reverse primer: 5′-ATTGCCTGGCACCGGAAAA-3′; GAPDH forward primer: 5′-TGTGGGCATCAATGGATTTGG-3′, reverse primer: 5′-ACACCATGTATTCCGGGTCAAT-3′; miR-3934-5p forward primer: 5′-GCCAGCTCCTACATCTCAGC-3′, reverse primer: 5′-AGCCTGACTTGCTAGTGGATTAT-3′; and U6 forward primer: 5′-CTCGCTTCGGCAGCACA-3′, reverse primer: 5′-AACGCTTCACGAATTTGCGT-3′.

### RNA-seq analysis

Total RNA extracted from the pre-isolated basophils using TRIzol reagent (Invitrogen) according to the manufacturer’s instructions. The quality of the RNAs was assessed using Nanodrop 2000 spectrophotometry (Thermo Fisher Scientific, Inc), after which the samples were sent to Vazyme Biotech (Nanjing, China) for RNA-sequencing.

### Bioinformatics, plasmid construction, and dual-luciferase reporter assay

The target of miRNA-3934 was obtained from starBase (Version 3.0) prediction. GenePharma synthesized a plasmid containing the sequences of miRNA-3934 paired with the RAGE 3′-UTR (RAGE-wild type (WT)) or mutant 3′-UTR (RAGE-mutant type (MUT)) regions. The miRNA-3934 mimics and RAGE (GenePharma) were co-transfected into 293 T cells. Luciferase activity was then examined 48 h after transfection using the dual-luciferase reporter assay kit (Beyotime, Shanghai, China).

### Flow cytometric apoptosis assay

Human basophils were collected after various treatments and stained with Annexin-V-FITC/PI following the manufacturer’s instructions. Analysis of the apoptotic and live cells was performed using FACSCalibur (BD Biosciences, San Jose, CA) flow cytometry. The scatter diagram of the FACS results were as follows: Q4, non-apoptotic cells (FITC−/PI−); Q3, apoptotic cells at early stage (FITC+/PI−); Q2, apoptotic cells at advanced stage (FITC+/PI+); Q1, mechanically injured cells (FITC−/PI+). The apoptosis rate was calculated as the sum of the ratio of apoptotic cells in Q3 + Q2.

### Western blot

Protease inhibitor cocktail was used to isolate total proteins, after which aliquots (30 μg/μl) were separated by SDS-PAGE and transferred to PDVF membranes. The membranes were then incubated with the primary antibodies (anti-RAGE, anti-Smad2, anti-p-Smad2, anti-Smad3, anti-p-Smad3, anti-p38, anti-p-p38, anti-Smad7 and anti-GAPDH, all purchased from Abcam, Cambridge, MA, USA) overnight at 4 °C. Glyceraldehyde-3-phosphate dehydrogenase (GAPDH) was used as a loading control. The following day, the membranes were incubated with the chemiluminescent reagent BeyoECL Plus and HRP-conjugated secondary antibodies (Beyotime, Shanghai, China). The signals were detected and photographed using a Tanon 6100 Chemiluminescent Imaging System (Tanon, Shanghai, China).

### Enzyme-linked immunosorbent assays

Commercially available ELISA kits (Nanjing Jiancheng Bio-Engineering Institute Co., Ltd, Nanjing, China) were used to determine the expression levels of IL-6, IL-8 and IL-33. The ELISAs were performed following the manufacturer’s protocols.

### Statistical analysis

Continuous variables are expressed as the median (interquartile range), and categorical variables are described as a number and percentage (%). Student’s t-test or analysis of variance (ANOVA) were used for data comparison. Apply parametric analysis to data with a sign and normal distribution. The diagnostic value of miR-3934 was calculated using ROC curve analysis. Pearson coefficient analysis was performed for the correlation analysis. Values of P < 0.05 were considered statistically significant. All statistical analyses were performed using GraphPad Prism v6.0.

## Results

### Genome-wide RNA sequencing to identify miRNAs differentially expressed in basophils from asthma patients and healthy controls

**Fig. 1:**
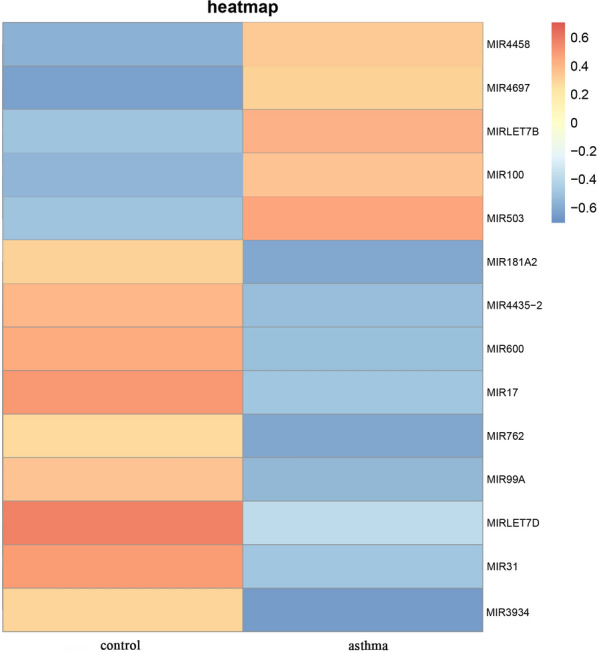
Heatmap showing expression of 14 differentially expressed miRNAs in asthmatic patients

**Fig. 2:**
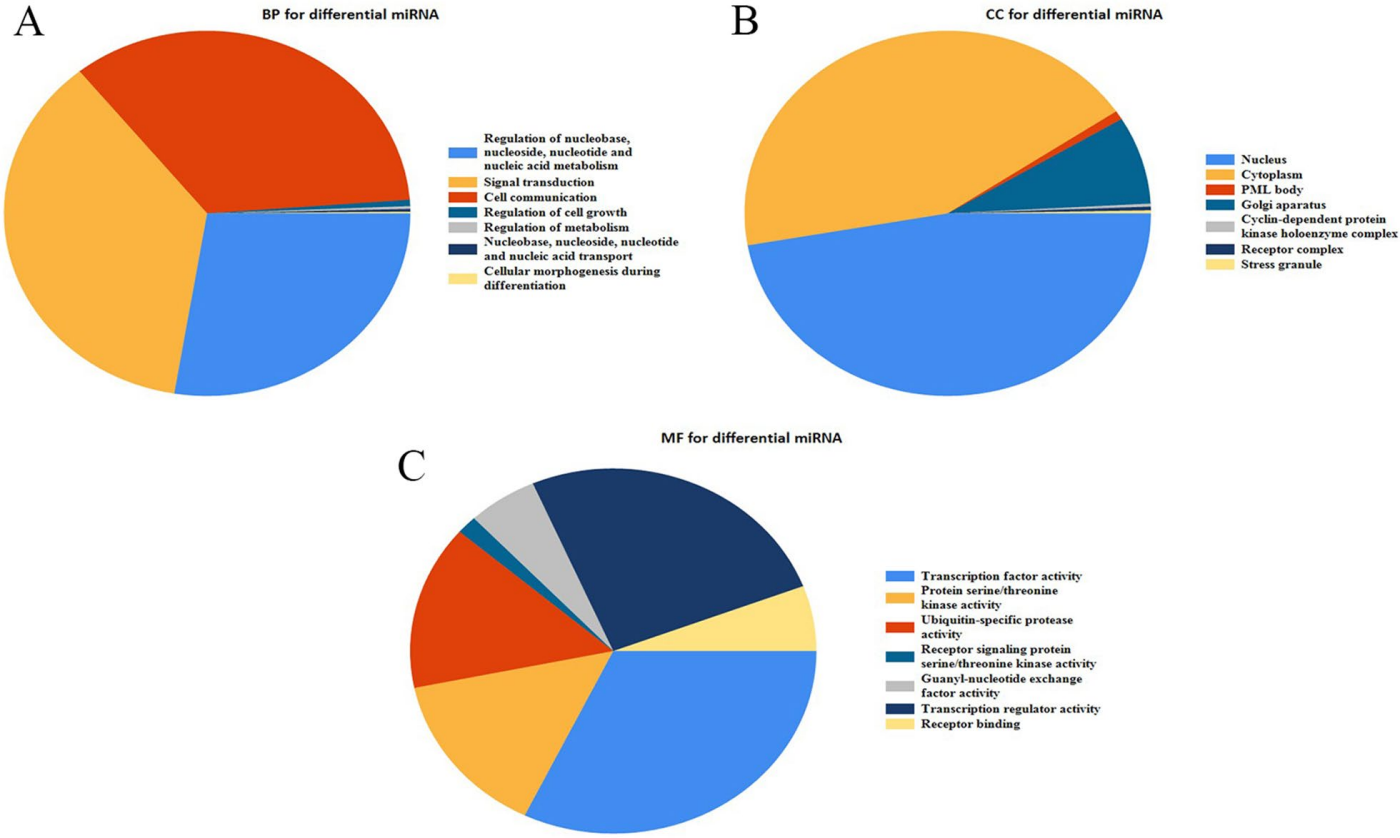
Gene ontology functional enrichment analysis of 14 key miRNAs. The relative expression levels are shown by the proportion of color. **A** Ontology source: biological process (BP). **B** Ontology source: cell component (CC). **C** Ontology source: molecular function (MF)

We first performed sequencing analysis to identify miRNAs differently expressed in basophils from asthma patients and healthy controls. As shown in Fig. 1, we detected significant alternations in the expression of 14 miRNAs, including 5 up-regulated and 9 down-regulated miRNAs. Results of a gene ontology (GO) analysis to determine the influence of miRNAs on the genome-wide expression of the genes are shown in Fig. 2 .There were no significant differences between the asthma and control groups with respect to age, gender or white blood cells count. However, there were differences in smoking history and allergy history. (Table 1, p > 0.05).

### Down-regulation of miR-3934 in peripheral blood mononuclear cells of asthma patients

**Fig. 3:**
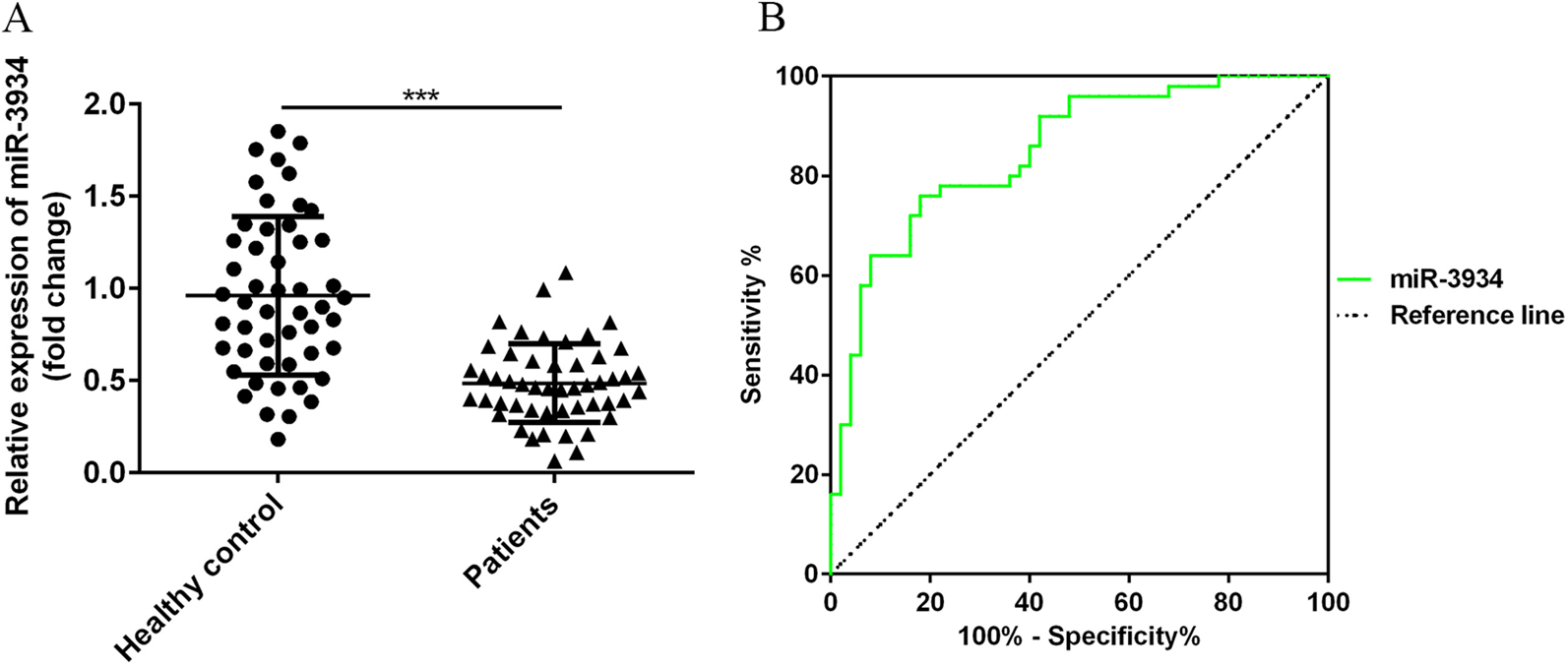
Potential diagnostic value for asthma of miR-3934 in peripheral blood mononuclear cells (PBMCs). **A** Down-regulation of miR-3934 in PBMCs from asthmatic patients. **B** Receiver operating characteristic (ROC) analysis showing miR-3934 can be used to distinguish between asthmatic patients and healthy controls. *** p < 0.01

Based on the sequencing results, miR-3934 was one of the miRNAs most down-regulated in basophils from asthmatic patients. To confirm that finding, we used RT-qPCR to compare expression levels of miR-3934 in peripheral blood mononuclear cells (PBMCs) from 50 asthma patients and 50 healthy volunteers. As shown in Fig. 3A, expression of miR-3934 was significantly lower in the asthma group than the control group (p < 0.01). The diagnostic value of miR-3934 was then evaluated using receiver operating characteristics (ROC) curve analysis. We compared asthma status with miR-3934 expression levels to generate curves. Then, we found that the area under curve (AUC) for miR-3934 was 0.8348 (95% CI 0.7544 to 0.9152, Fig. 3B), indicating that miR-3934 levels are a sensitive biomarker able to distinguish asthma patients from healthy individuals.

### RAGE is a target of miR-3934 in asthma

**Fig. 4:**
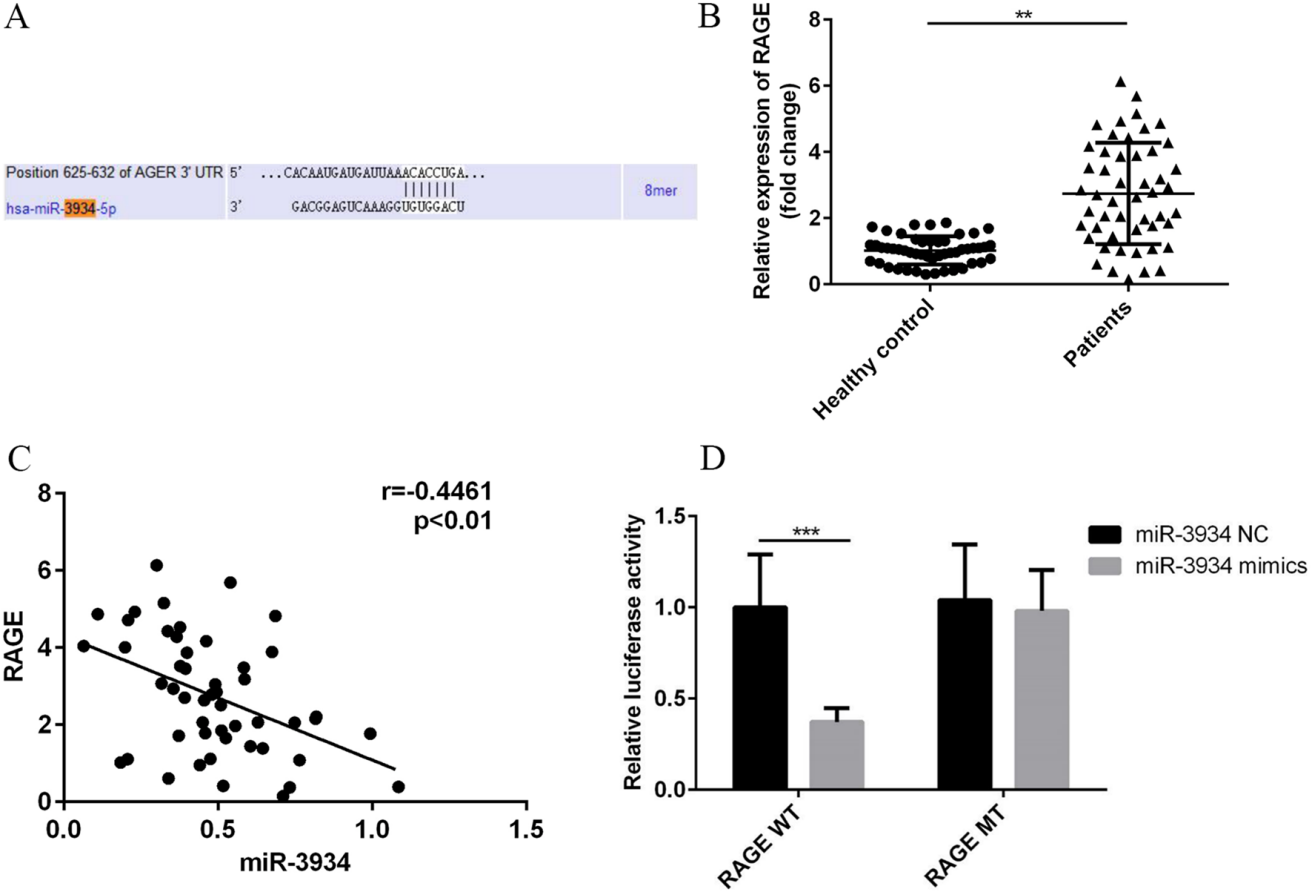
Relationship between miR-3934 and RAGE in asthma. **A** Targetscan prediction result for miR-3934. **B** Comparison of miR-3934 expression levels in PBMCs from asthmatic patients and healthy controls. **C** Correlation between miR-3934 expression and RAGE expression in asthmatic patients. **D** Effects of transfecting miR-3934 mimics on the luciferase activity driven by RAGE wild-type (WT) and mutant type (MUT). ** p < 0.01 *** p < 0.01

miRNAs are known to exert their functions via targeting mRNAs [13]. The bioinformatic analysis (Targetscan) identified RAGE as a target of miR-3934 (Fig. 4A). To investigate the relationship between miR-3934 and RAGE in asthma, RT-qPCR was performed to assess RAGE expression in PBMCs. We found that RAGE expression was significantly higher in PBMCs from asthma patients than from the healthy controls (Fig. 4B, p < 0.01) and that miR-3934 expression correlated negatively with the RAGE expression (Fig. 4C, p < 0.01). Moreover, dual-luciferase reporter assays confirmed a direct relationship between miR-3934 and RAGE. Transfection of miR-3934 mimics notably decreased luciferase activity driven by wild-type (WT) RAGE group but not a RAGE mutant (Fig. 4D < 0.01). These results suggest that miR-3934 may be involved in the basophil changes in asthma through its regulation of RAGE expression.

### miR-3934 inhibits AGE-induced basophil apoptosis *by *targeting RAGE in vitro

**Fig. 5:**
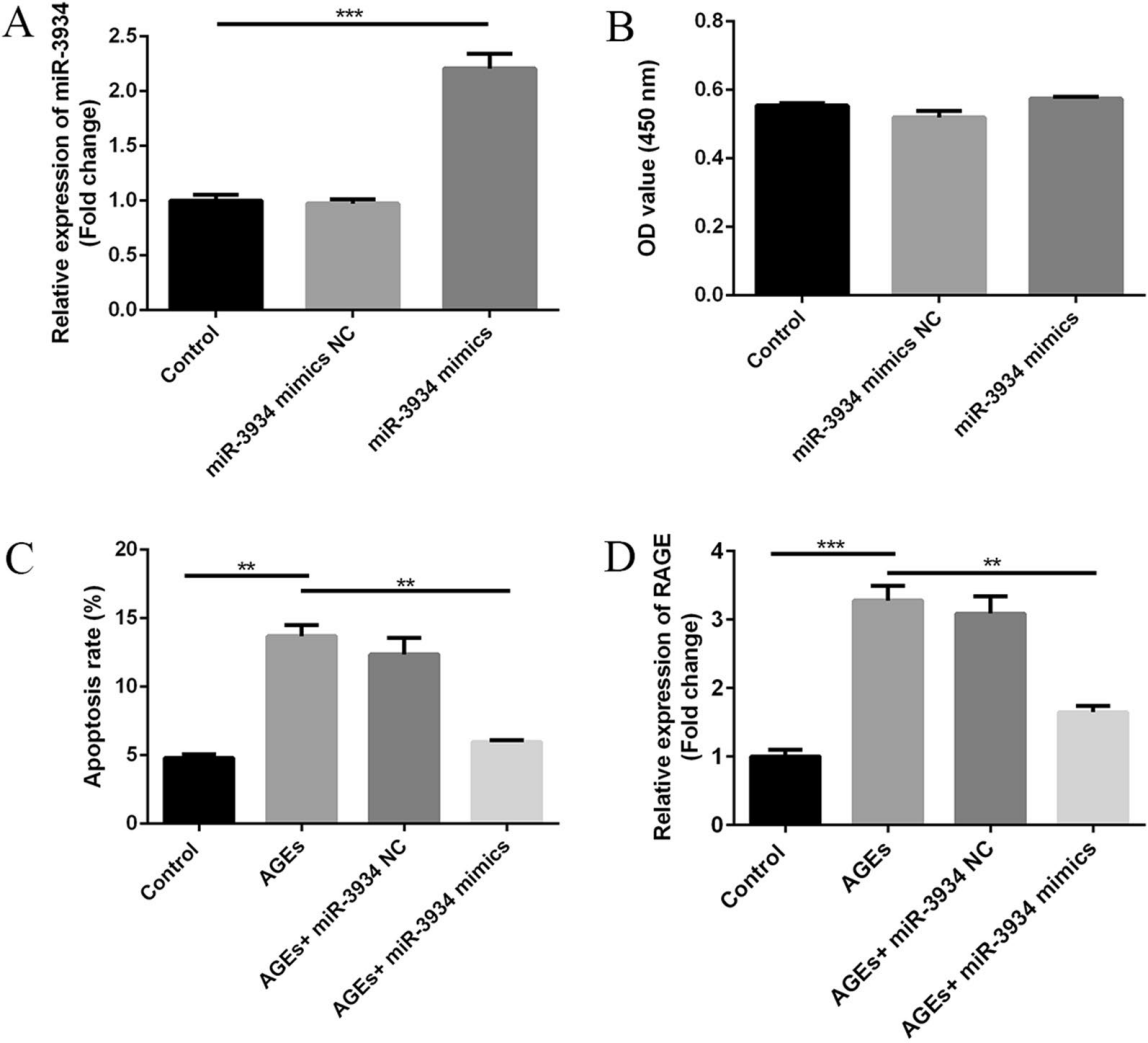
Effect on basophil apoptosis of miR-3934-mediated inhibition of advanced glycation end products (AGEs) secondary to its suppression of RAGE expression. **A** Effects of miR-3934 mimics transfection on levels of miR-3934 expression in basophils. **B** Effects of AGEs treatment and miR-3934 mimics transfection on basophil proliferation. **C** Effect of AGEs treatment on apoptosis levels among basophils. **D** Effects of AGEs treatment on levels of RAGE expression. *** p < 0.01 **p < 0.01

We observed that transfection of miR-3934 mimics, but not miR-3934 NC, markedly increased expression of miR-3934 in basophils (p < 0.01) (Fig. 5A). After basophils transfected with miR-3934 mimics or NC were treating with AGEs, CCK-8 cell proliferation assays as well as flow cytometric assays indicated that neither AGEs nor miR-3934 mimics had a notable effect on basophil proliferation of (Fig. 5B, p > 0.05). On the other hand, AGEs induced a marked increase in apoptosis among basophils (Fig. 5C, p < 0.01) and increased expression of RAGE (Fig. 5D, p < 0.01). Transfection of miR-3934 mimic partially reversed the AGE-induced increase in the basophil apoptosis by inhibiting RAGE expression (Fig. 5C, D, p < 0.01).

### miR-3934 inhibits AGE-induced increases in the secretion of pro-inflammatory cytokines in basophils by targeting RAGE in vitro

**Fig. 6:**
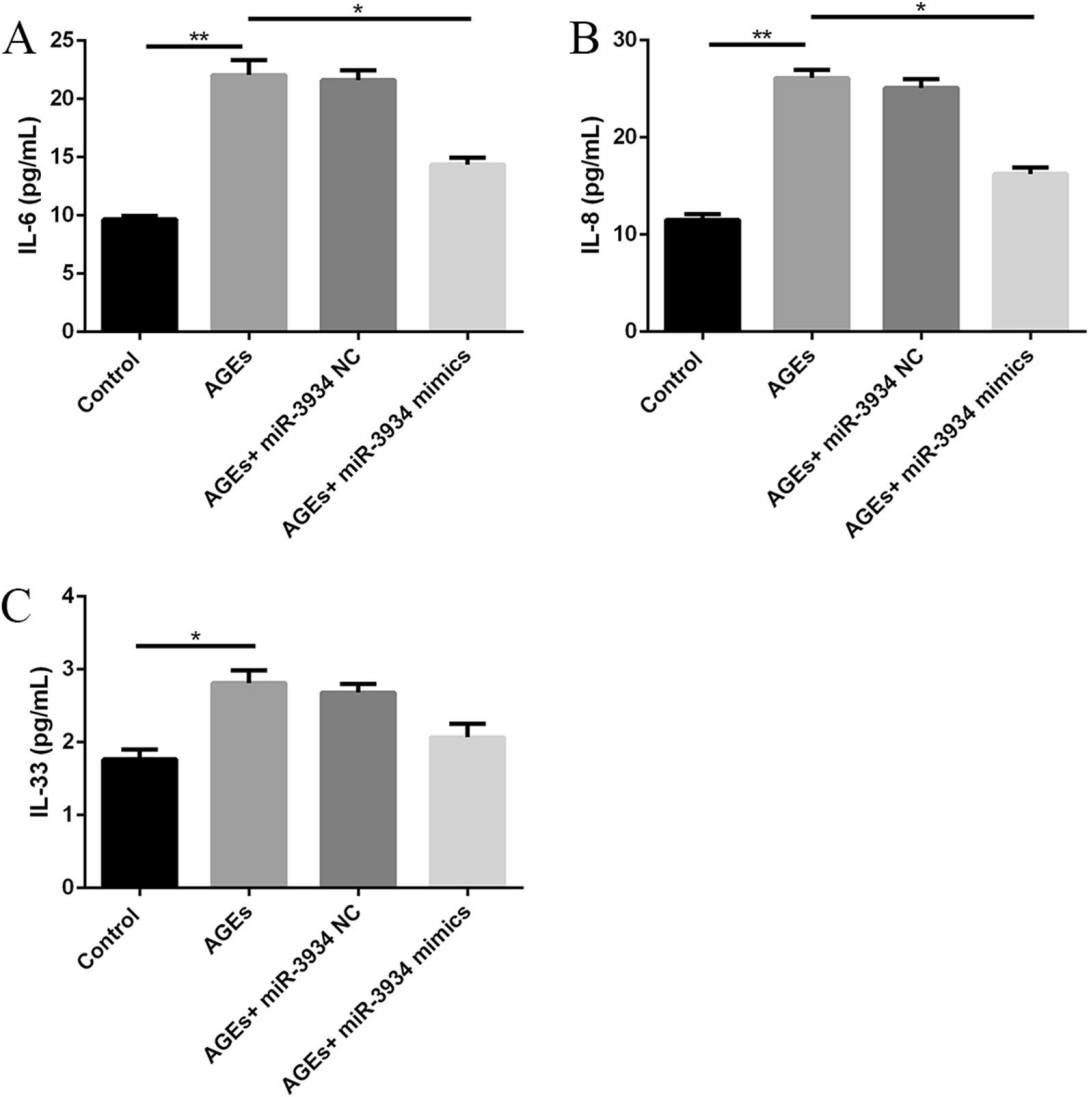
**:** Effects of miR-3934 mimics on the expression of pro-inflammatory cytokines in AGEs-treated basophils. **A** IL-6. **B** IL-8. **C** IL-33. *p < 0.01 **p < 0.01

Using ELISAs, we also assessed the effects of miR-3934 on the expression of the pro-inflammatory cytokine IL-6, IL-8 and IL-33 in AGE-treated basophils. We observed that expression levels of IL-6, IL-8 and IL-33 were all markedly increased in AGE-treated basophils, and that effect was partially reversed by miR-3934 mimics (Fig. 6, p < 0.01).

### miR-3934 inhibits AGE-induced activation of the TGF-β/Smad signaling pathway

**Fig. 7:**
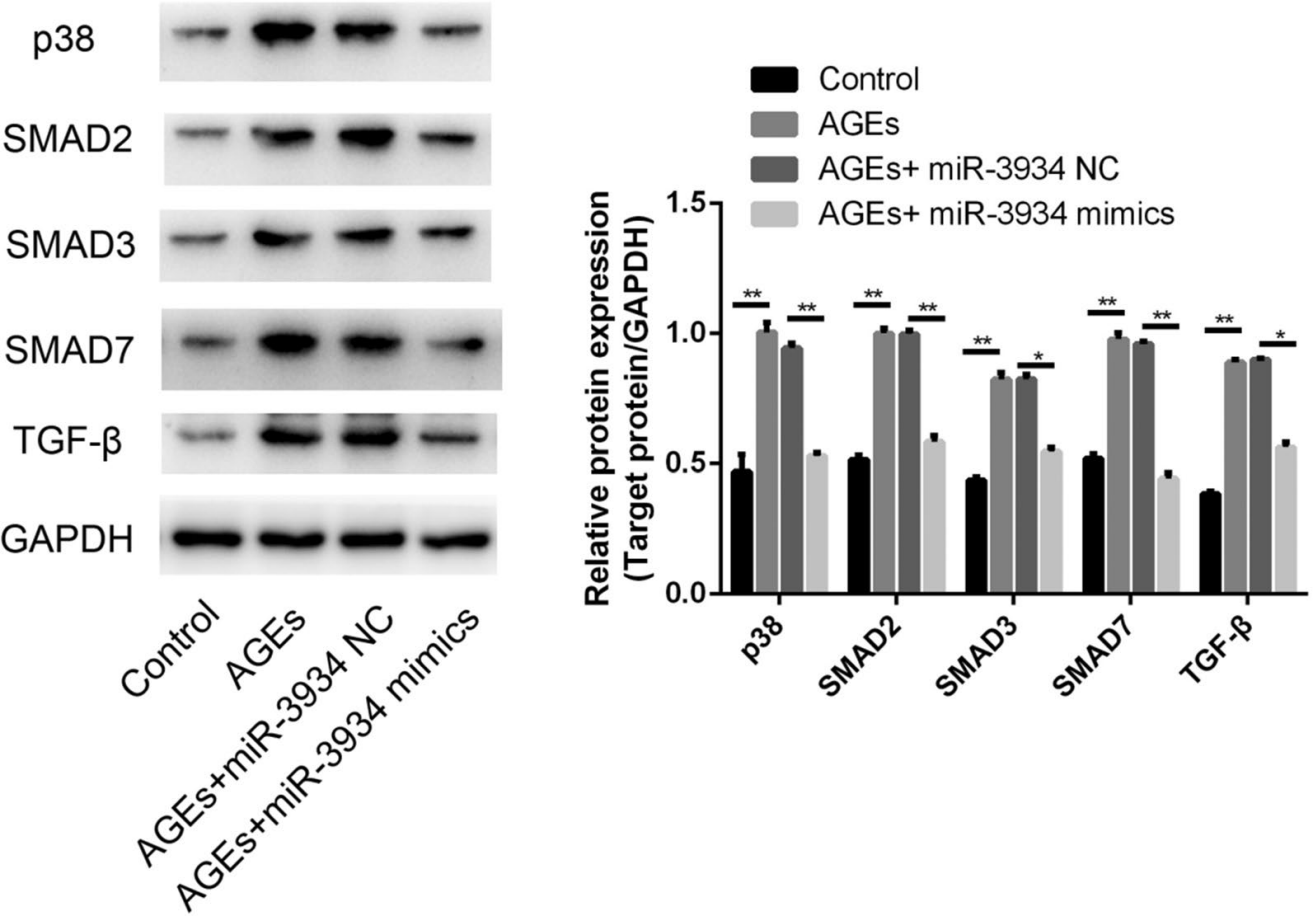
miR-3934 inhibits AGE-induced activation of TGF-β/Smad signaling. Expression of TGF-β/Smad signaling molecules were examined using Western blot method. *p < 0.05, **p < 0.01

To reveal how miR-3934 is involved in basophil changes in asthma, expression of key molecules in the TGF-β/Smad signaling pathway was examined. We found that, within basophils, AGEs mediate activation of TGF-β/Smad signaling by increasing expression of TGF-β and Smad7 as well as phosphorylation of Smad2, Smad3 and p38 (Fig. 7, p < 0.01). miR-3934 mimics partially blocked AGE-induced activation of the TGF-β/Smad signaling (p < 0.01).

### TGF-β blocks miR-3934-induced anti-apoptotic and anti-inflammatory effects on basophils

**Fig. 8:**
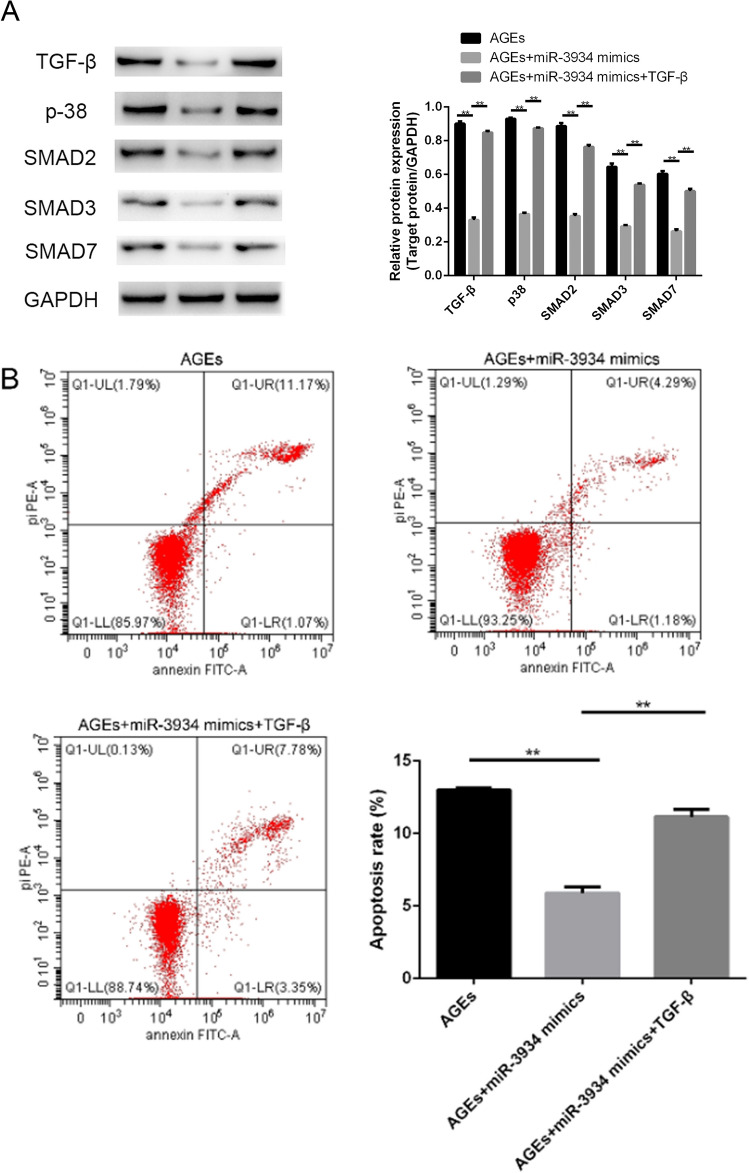

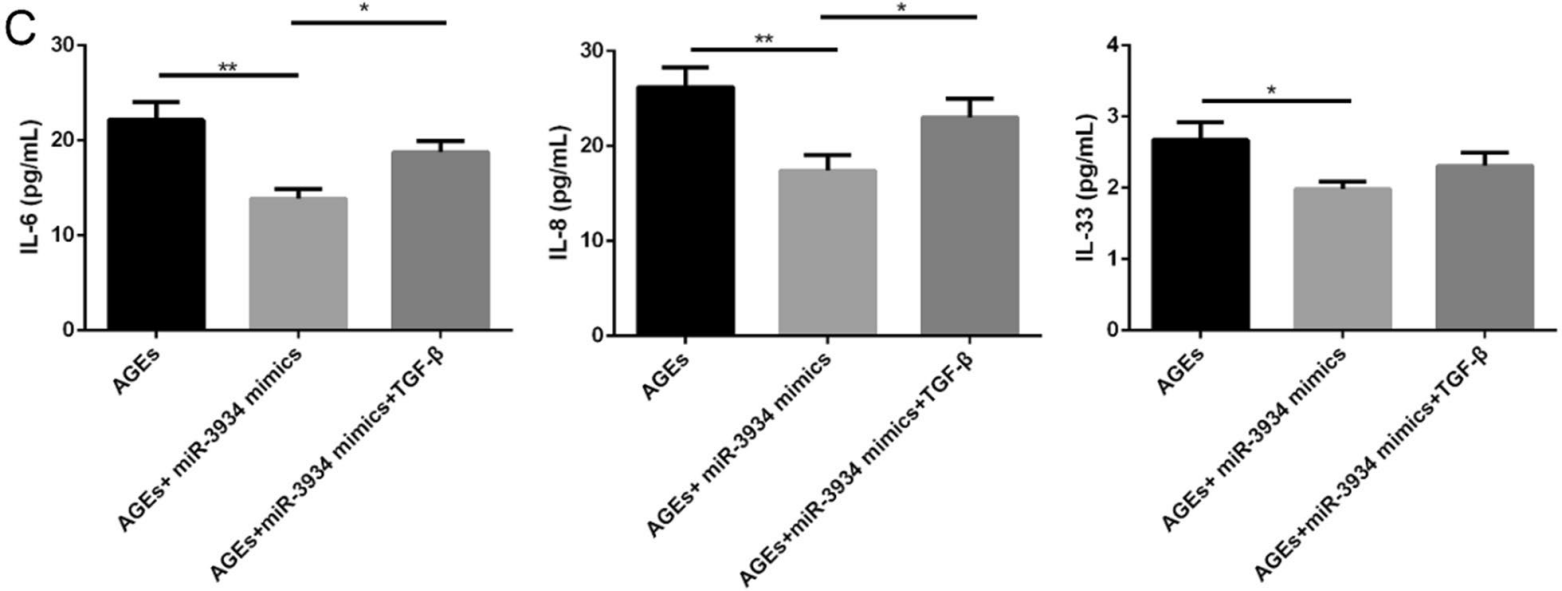
TGF-β partially blocked the effects of miR-3934 mimics on basophils. **A** Western blot analysis of basophils subjected to the indicated treatments. **B**. Flow cytometric analysis of apoptosis among basophils subjected to the indicated treatments. **C**. Levels of IL-6, IL-8 and IL-33 in cell culture supernatants from basophils subjected to the indicated treatments. *p < 0.05, **p < 0.01

To further assess the protective effects of miR-3934 regulating basophils against asthma progression via targeting RAGE and TGF-β/Smad signaling, miR-3934-transfected basophils were treated with TGF-β, after which apoptosis and secretion of inflammatory cytokines were examined. We observed that, in miR-3934-treated basophils, TGF-β significantly increased the expression of Smad7 and phosphorylation of Smad2, Smad3 and p38 in (Fig. 8A, p < 0.01) and also contributed to increases in apoptosis and secretion of IL-6, IL-8 and IL-33 (Fig. 8B, C, p < 0.01).

## Discussion

In this study, we observed that miR-3934 was down-regulated in basophils from patients with asthma and, more importantly, that miR-3934 suppresses RAGE expression and, in turn, AGE-induced TGF-β/Smad signaling. This suggests miR-3934 likely plays a protective role in asthma.

Dysregulation of miRNAs in asthma has been observed in many previous studies. For example, miR-218-5p reportedly targets δ-catenin to contribute to eosinophilic airway inflammation in asthma [26]. In addition, miR-145-5p promotes asthma occurrence via 3A (an inhibitory kinesin family member) within airway epithelial cells [27]. And miR-23a is reportedly involved in the pathogenesis of asthma by targeting BCL2 in airway epithelial cells and CXCL12 in fibroblasts [28]. It remained unclear, however, whether miRNA expression is altered in basophils of patients with asthma. To address that issue, we first performed RNA sequencing to identify miRNAs differentially expressed in basophils from asthma patients and healthy controls. This revealed 12 miRNAs that were up-regulated and 9 that were down-regulated in asthma patients as compared to healthy controls. In addition, GO functional enrichment analysis revealed that those miRNAs likely participate in the metabolic process, cell proliferation, cellular response to chemical stimulus, positive regulation of cellular process and biological process of basophils.

Based on the sequencing results, it appears miR-3934 is greatly down-regulated in asthma patients. Most earlier studies of miR-3934 investigated its role in cancer. For example, it was reported that miR-3934 is up-regulated in colorectal cancer cells [29] and that down-regulation of miR-3934-5p may enhance the sensitivity of A549 non-small cell lung cancer cells to cisplatin by targeting TP53INP1 [30]. Here, we observed that miR-3934 is significantly down-regulated in PBMCs from asthma patients, and results of ROC analysis showed that miR-3934 is a sensitive biomarker that can be used to distinguish asthma patients from the healthy volunteers, suggesting it could potentially serve as a diagnostic marker.

miRNAs exert their actions by inhibiting translation of target mRNAs or by directly cleaving them [13, 31]. Using bioinformatics, we found that RAGE is a target of miR-3934 in basophils. RAGE is a 35 kDa protein in the immunoglobulin superfamily, and its increased expression can lead to inflammatory conditions [23, 32]. In the case of asthma, the close relationship between RAGE overexpression and asthma development has been reported previously by ourselves and others [22, 23, 33, 34]. In the present study, we observed that RAGE is up-regulated in PBMCs from asthma patients, which was consistent with its previously reported expression pattern [22]. In addition, we confirmed both the negative correlation between miR-3934 and RAGE expression and the direct relationship between miR-3934 and RAGE. These experimental results suggest that by targeting RAGE, miR-3934 may play a protective role during asthma development.

We previously reported that AGEs can mediate increased expression of RAGE in basophils, thereby promoting apoptosis and increasing secretion of inflammatory cytokines [22]. Interestingly, in the present study, we noticed that adding miR-3934 to AGE-treated basophils significantly decreased RAGE expression while inhibiting AGE-induced apoptosis and increases in secretion of pro-inflammatory cytokines. This suggests the protective role of miR-3934 during the development of asthma reflects its inhibitory effects on basophil apoptosis and secretion of pro-inflammatory cytokines mediated through targeting RAGE.

TGF-β/Smad is an essential signaling pathway in the pathogenesis of asthma [9, 35, 36]. Previous studies showed that downstream TGF-β/Smad signaling activated by RAGE leads to basophil apoptosis and inflammatory conditions in asthma [37]. We explored whether miR-3934 exerts its anti-apoptotic and anti-inflammatory effects by inhibiting TGF-β/Smad signaling downstream of its RAGE suppression. As expected, miR-3934 mimics partially inhibited AGE-induced activation of TGF-β/Smad signaling and, interestingly, addition of TGF-β to miR-3934 mimics-treated cells increased apoptosis and secretion of the inflammatory cytokines. In other words, if we can increase the expression of miR-3934 in asthma patients, the expression of RAGE can be inhibited, thereby reducing the activation of the TGF-β/Smad pathway, and ultimately reducing the development of asthma. miR-3934. This means that miR-3934 may play a protective role in asthma.

## Conclusion

These results indicate that miR-3934 acts to mitigate the pathogenesis of asthma by targeting RAGE and, in turn, suppressing TGF-β/Smad signaling. Moreover, they provide novel evidence for the potential application of miR-3934 as a diagnostic marker for early diagnosis of asthma.

## Data Availability

The datasets used and analysed during the current study are available from the corresponding author on reasonable request.

## Abbreviations

ROC: Receiver operating characteristics
AGEs: Advanced glycation end products
RAGE: Receptor for advanced glycation end products
miRNA: MicroRNA
PBMCs: Peripheral blood mononuclear cells
NC: Negative control
WT: Wild type
MUT: Mutant type
GAPDH: Glyceraldehyde-3-phosphate dehydrogenase
